# Quantifying the Effects of Network Latency for a Teleoperated Robot

**DOI:** 10.3390/s23208438

**Published:** 2023-10-13

**Authors:** Adriana Noguera Cundar, Reza Fotouhi, Zachary Ochitwa, Haron Obaid

**Affiliations:** 1 Department of Mechanical Engineering, University of Saskatchewan, Saskatoon, SK S7N 5A9, Canada; apn252@usask.ca (A.N.C.); zachary.ochitwa@usask.ca (Z.O.); 2 Department of Medical Imaging, University of Saskatchewan, Saskatoon, SK S7N 0W8, Canada; haron.obaid@usask.ca

**Keywords:** robotic assisted, robotic tele-ultrasound, time delay, network latency, communication signal

## Abstract

The development of teleoperated devices is a growing area of study since it can improve cost effectiveness, safety, and healthcare accessibility. However, due to the large distances involved in using teleoperated devices, these systems suffer from communication degradation, such as latency or signal loss. Understanding degradation is important to develop and improve the effectiveness of future systems. The objective of this research is to identify how a teleoperated system’s behavior is affected by latency and to investigate possible methods to mitigate its effects. In this research, the end-effector position error of a 4-degree-of-freedom (4-DOF) teleultrasound robot was measured and correlated with measured time delay. The tests were conducted on a Wireless Local Area Network (WLAN) and a Virtual Local Area Network (VLAN) to monitor noticeable changes in position error with different network configurations. In this study, it was verified that the communication channel between master and slave stations was a significant source of delay. In addition, position error had a strong positive correlation with delay time. The WLAN configuration achieved an average of 300 ms of delay and a maximum displacement error of 7.8 mm. The VLAN configuration showed a noticeable improvement with a 40% decrease in average delay time and a 70% decrease in maximum displacement error. The contribution of this work includes quantifying the effects of delay on end-effector position error and the relative performance between different network configurations.

## 1. Introduction

Teleoperated control is the functionality for a user to manipulate a device remotely despite vast distances. Robots which can be controlled remotely are valuable, as they can be used in place of humans—whether for safety, accessibility, or capability. As a result, the use of teleoperated robotic systems has increased over past decades [1,2]. Applications of teleoperated systems include construction, space, manufacturing, submarine exploration, and the field of medicine [3,4]. Within medicine, teleoperated systems can be used for procedures such as ultrasound imaging or minimally invasive surgery [5,6]. Considering ultrasound imaging specifically, tele-robotics can help a specialist diagnose patients remotely, without the need for in-person meetings. This ability is crucial because of the possible pandemic situation or the need to reach patients in isolated locations. However, extra caution must be taken in medicine for safety and reliability in the human–robot interaction. This is especially true for surgery, where precision is crucial [7]. The development of precise and easy-to-handle teleoperated systems is imperative to contribute to the improvement of healthcare services. 

Teleoperated robots are often employed in real-time. Combined with the safety needed for medical applications, it is necessary to ensure a high Quality of Service (QoS). In this case, QoS is the overall quality of the communication between the robot and the operator. Delay, jitter, or packet loss will be passed onto the robot and cause undesired behavior. For example, packet loss in a command to move may cause the robot to stutter or not move at all [1]. Although the different aspects of QoS can influence the operation of teleoperated robots, this research is focused on how delay affects the position accuracy of a manipulator.

The basic structure of a teleoperated system consists of three main parts: a master station, a slave station, and a communication channel. In the master station, there are tools to perform tasks, such as the controller, a computer, a monitor, and the operator. On the other hand, the slave station is composed of a manipulator, a probe attached to the end-effector of the manipulator, the patient, and a medical assistant. The communication channel transmits data, images, and positions. Because of the great distances between master (radiologist) and slave (patient) stations, time delay in sending and receiving signals is a concern in teleoperated systems. This time delay may cause instability and alters the behavior of the system [8]. This delay has been shown to negatively affect operators’ experience of the procedure [9,10].

There are several control methods, each with a trade-off in complexity and robustness. In the case of teleoperation, the control system is often designed to mitigate the effects of delay. However, the behavior of delay must be well understood to best mitigate it or to strike a balance between delay mitigation or another factor, such as responsiveness. Examples of such systems are predictive controllers such as the Bilateral Generalized Predictor Controller [11]. Here, a constant time delay is used to estimate the future position of the end-effector, and it can thus affect the performance of the controller. Alternatively, some controllers have been developed to actively update the value of delay for optimal delay mitigation [12,13]. However, this may cause issues or undesired complexities when there are several other control objectives like disturbance rejection [14]. Therefore, in some cases, it is more practical to use a well-chosen value of delay based on its behavior and effects instead of a complex control system. Another strategy to prevent complications from latency is to set the computer to “jump in” and prevent unsafe motion during excessive delay [15,16].

Of course, the most effective method to reduce error from delay is to reduce the delay itself. However, it is also important to quantify the effects of delay and how it may affect a teleoperated system. To select an appropriate network and control method, a designer must be aware of the relative performance of different network configurations as well as how network delay may affect their systems. Therefore, the objectives of this research are to: (1) quantify the latency in a teleoperated system and how it affects position accuracy, and (2) investigate how changing the network configuration can alter the latency in the system. 

## 2. Materials and Methods

### 2.1. MSK Robotic System

The MSK (musculoskeletal) telerobotic system is a remotely controlled ultrasound imaging manipulator developed by the Robotics Laboratory at the University of Saskatchewan [5]. It is designed for the ultrasound imaging of the upper and lower limbs. This system consists of three main parts: the master station, the slave station, and the communication network. The master station is responsible for remotely commanding the robot by sending position commands and providing audio and visual feedback, such as videos of the patient and ultrasound, to the technician. In the master station, there is a joystick used by a specialist to move the robot manipulator and a computer that enables a connection with the manipulator. 

**Figure 1:**
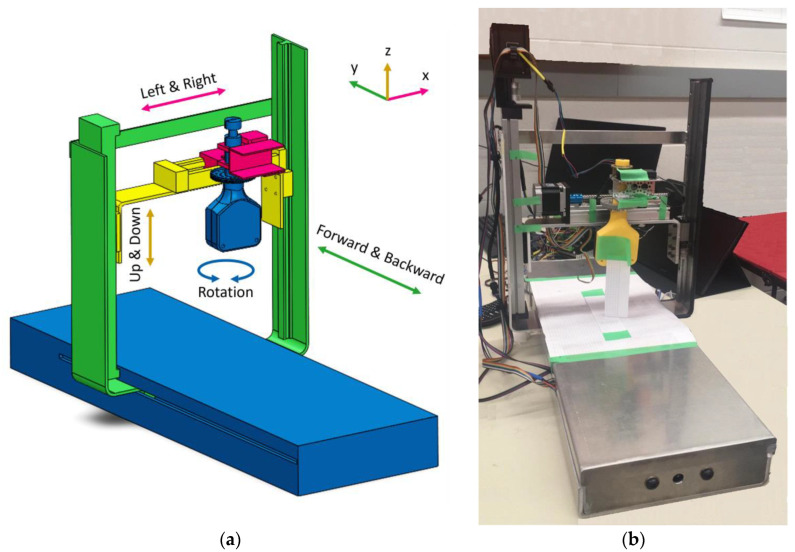
(**a**) MSK robot manipulator, and (**b**) Experimental setup.

The slave station, shown in Figure 1, contains a manipulator that receives the joystick signals from a user. The manipulator is a 4-degree-of-freedom (DOF) robot with three prismatic joints and one rotational joint. The 4 DOFs are defined as horizontal (along axis *X*), horizontal (along axis *Y*), vertical (along axis *Z*), and rotation (around axis *Z*), which holds an ultrasound probe. Each degree of freedom is independently driven by stepper motors. Analysis of the manipulator, including the Denavit–Hartenberg (DH) parameters, kinematic analysis, and dynamic analysis can be found in Appendix A, Appendix B, and Appendix C, respectively. The slave station also has a video connection to the master station, which allows the patient to communicate with the operator. 

The communication network is responsible for the transmission of information between master and slave stations. This communication network enables the user to execute remote control by connecting both stations through WLAN or Wi-Fi. Both the master and slave stations were connected through the same wireless network during tests. Other users or services were working in the same network, reducing its speed. The network speed was measured to range from 0.72 to 2.42 megabits per second. The control signal is encoded to ASCII with an average and maximum size of 13 and 23 bytes per package, respectively. This package is then sent via Transmission Control Protocol (TCP). The wireless networking equipment complies with the IEEE 802.11n standard.

The MSK device is employed in real-time procedures; therefore, it is highly reliant on the quality of the communication channel. If the required quality of service is not met, the whole functionality undergoes changes. Examples of these changes include the emergence of delay, jitter, and packet loss, which are translated into the undesired execution of the manipulator. The undesired parameters may reduce accuracy in the motion of the MSK device, which is important for good image quality and the safety of the patient.

### 2.2. Time Delay Measurement

An objective of this research was to measure the time delay and corresponding manipulator’s behavior, such as the end-effector’s position error, in a teleoperated system. The time delay is the amount of time it takes for the control signal to be sent from the master station to when the slave station receives the signal and initiates the response. In this study, time delay was measured using two methods: a computer program and a video recording. The computer program (Wireshark) measured the time for an information packet to be sent from the master station and received at the slave station. This was used to measure the overall time delay between networks. While the computer program was relatively precise, its output could not be correlated to specific commands. The second method, video recording, was used to verify the former method, as well as measure the response time for each discrete command. This was performed by recording both the controller and MSK device together. By counting the frames between input and motion, the delay time was measured.

Both delay measurement methods were initiated simultaneously over a series of trials. In these trials, only 1 DOF was operated at a time, with the manipulator coming to rest in between each input to clearly distinguish movement for the video recording. In the end, the experiment consisted of 74 movements using all four degrees of freedom. 

### 2.3. Position Error Measurement

To measure the time delay, experimental tests were performed to determine the presence of delay in the system. Each movement was captured on video at 30 frames per second (FPS) from lateral and front views of the manipulator. The videos were assumed to be consistent and were used to measure time delays. Delay time was obtained by measuring the elapsed time from when the joystick button was pressed to when the robot had executed its action. A chronometer was used to measure travel time for each segment while a caliper measured the position of the end-effector.

**Figure 2:**
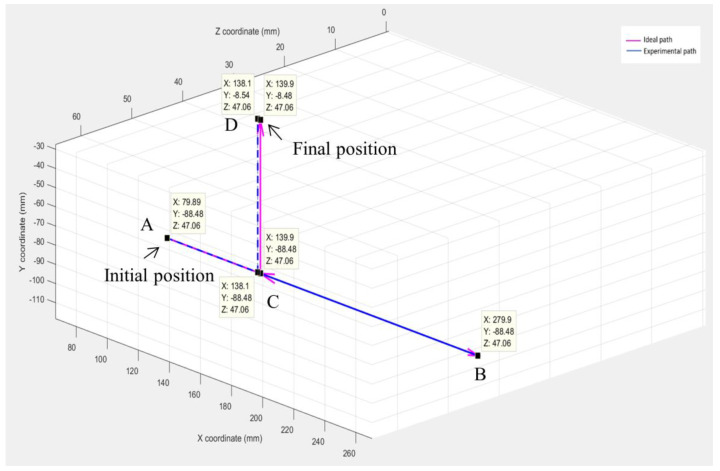
Experimental predefined path which goes through points A, B, C and D.

The next step was to measure how the behavior of a teleoperated device correlates with time delay. For this experiment, five pre-defined paths, like the path shown in Figure 2, were performed on the MSK robotic system. These five paths varied in length, direction of motion, and the number of times the direction changed. However, only 1 DOF was operated at a time.

In between different segments of a path, such as points B, C, and D in Figure 2, the manipulator would come to rest, and the position of the end-effector would be measured. This was performed by measuring the end-effector position in all three axes using calipers as shown in Figure 1b. This experimental position was then compared to the expected position to calculate the position error. This procedure was conducted at least five times on each path to check for repeatability.

### 2.4. VLAN Experiments

The second objective of this research is to investigate how network configurations may affect the behavior of a teleoperated robot. Originally, the MSK device was designed to be controlled over the same network using a WLAN connection. However, another configuration is a Virtual Local Area Network (VLAN). This configuration is similar to WLAN, except that the master and slave stations are partitioned into a dedicated channel and isolated from other network devices. The time delay and position error experiments were repeated with this setup and compared with the previous results to see if there is a noticeable change when employing the VLAN configuration.

## 3. Results

### 3.1. WLAN Time Delay

**Figure 3:**
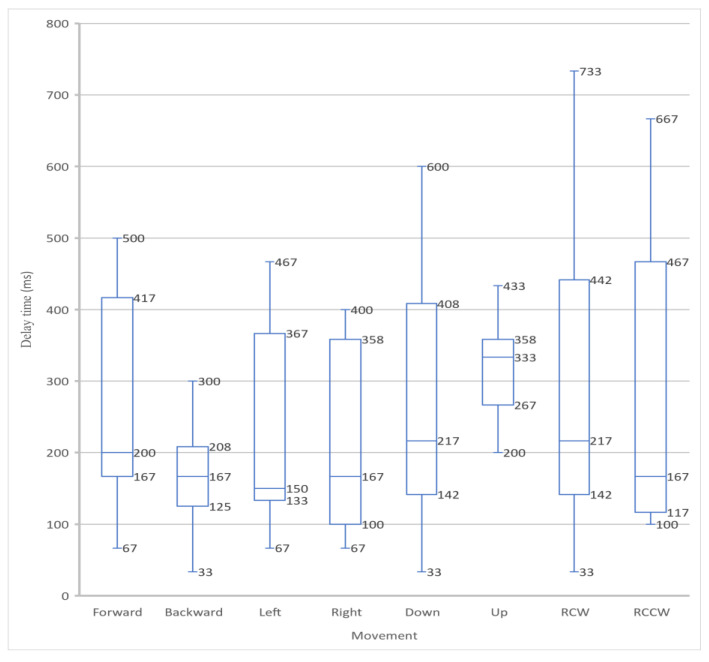
Delay per control input.

After conducting the time-delay experiment, a box and whisker plot was generated from the acquired data. This statistical method was used due to its capability of pattern identification and easy visual interpretation of data. Figure 3 presents a plot of delay information where the data are grouped according to the movements performed. Forward and backward are motions in the positive and negative *y*-axis, respectively. Left and right are motions in the positive and negative *x*-axis, respectively. Up and down are motions in the positive and negative *z*-axis, respectively. Finally, RCW and RCCW are rotation clockwise and rotation counterclockwise about the z-axis, respectively.

In Figure 3, the data are divided into quartiles. This graph provides more information about the distribution of delay data present in the MSK system. Most control inputs show a larger range of values between the second quartile, or median, and the third quartile. Nearly all inputs had a median time delay of around 200 ms except for upward motion, which had a median of 333 ms. According to Figure 3, the ranges of time delay are between a minimum of 33 ms and a maximum of 733 ms. Note that with a video capture speed of 30 FPS, the lowest possible delay time is 33 ms. According to [9,17,18], the maximum time delay is an important factor in ensuring a safe, high-quality experience and should be limited to around 300–400 ms. Therefore, using the WLAN network configuration used in the experiment, the measured delay was found to be at the upper limit and should thus be improved.

While the network analyzer software could not distinguish between the different types of commands, it had a higher sampling rate and resolution. The software showed that most delay values were concentrated in a range of 225 to 375 ms. This communication channel also exhibited occasional spikes of up to 750 ms. One possible source of error which accounts for differences between motions relates to the limited video resolution and how the device was positioned relative to cameras. The revolute joint moves slower than the other joints and thus may have to move more to be detected by the camera. Similarly, the degree of freedom of moving “in” and “out” of the monitor would have a less perceived motion than the other prismatic joints.

### 3.2. WLAN Position Error

During tests, the end-effector position of the MSK manipulator was tracked experimentally and compared with the desired path (zero-delay). The position error was calculated using two different methods. First, the one-dimensional error, ∆, was calculated for every segment of the path using the formula

**Figure 4:**
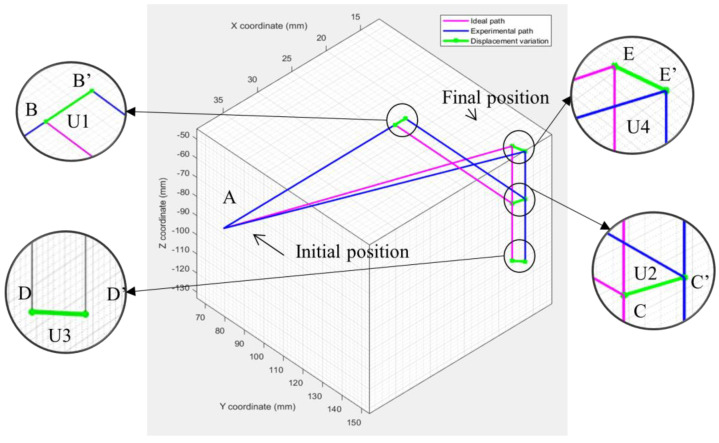
Position error calculation for each section for a path through points A, B, C, D, and E. B’, C’, D’, and E’ are the experimentally measured coordinates while U1, U2, U3 and U4 are the respective position errors.

(1) Δ=TDI−TDE

where *TDI* is the ideal travel distance and *TDE* is the experimental travel distance. This error is independent of any changes in direction. The second method is overall end-effector displacement, *W*, in a 3D space, which is calculated as:

(2) $$W=\sqrt{{\left(x_{IDE}-x_{EXP}\right)}^{2}+{\left(y_{IDE}-y_{EXP}\right)}^{2}+{\left(z_{IDE}-z_{EXP}\right)}^{2}}$$

where *x*, *y*, and *z* are the coordinates in their respective axes. The *IDE* subscript denotes the ideal coordinates while the *EXP* represents the experimental, or physical, coordinates. This type of position error is dependent on the trajectory chosen. The position error was calculated for each section as shown in Figure 4. The ideal position is denoted by the base letter, such as B, while the experimental position is denoted by an apostrophe, such as B′. W is the difference in position in three-dimensional space.

**Figure 5:**
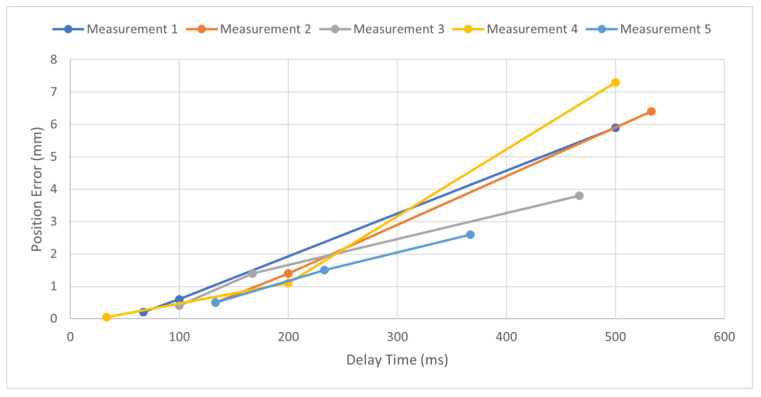
Delay time verses displacement error for five independent measurements.

This error calculation was performed at least five times for each of the five trajectories. Figure 5 shows five sample measurements taken for a single trajectory. While the measured delay may vary between measurements, each measurement showed a strong positive relationship between delay time and position error. This correlation can be calculated using Pearson’s correlation coefficient, which measures the linear correlation between two sets of data [19]. A coefficient value of +1 represents a perfect positive correlation while a coefficient value of −1 represents a perfect negative correlation. A value of ±1 means that all the data lie in a straight line while a value of 0 represents no correlation. The coefficient can be calculated using the equation:

(3) $$C\left(X,Y\right)=\frac{\sum \left(x-\bar{x}\right)\left(y-\bar{y}\right)}{\sqrt{\sum {\left(x-\bar{x}\right)}^{2}\sum {\left(y-\bar{y}\right)}^{2}}}$$

where *x* and *y* are the data sets for the delay time and position error in this experiment. $\bar{x}$ and $\bar{y}$ are the mean values of those data sets, respectively. Using this equation, the correlation coefficient was calculated to be +0.918, an indication that the two sets are strongly correlated if they are linear.

Despite a strong positive correlation between the delay time and position error, there were a few outlier points in individual measurements that did not follow the trend. For example, there were a couple of occasions when the data point with the largest delay time had a relatively small position error. Future studies can consider a large number of operations and trials to study the variance of position error across a range of delay times.

While delay time and position error had a strong correlation with each other, they did not have a strong correlation with travel distance, with a correlation coefficient of 0.293 and 0.331, respectively. These were calculated using Equation (3). Table 1 shows the average position and elapsed time over five measurements of a single trajectory.

As mentioned previously, TDI is the ideal travel distance, TDE is the experimental travel distance, ∆ is the one-dimensional position error, and W is the overall position error in 3D space. DT is the delay time measured between the desired and experimental trajectories. All trajectories showed the same general behavior: as the delay time goes up, so does the absolute position error. However, there was no clear pattern for when the experimental travel length was longer or shorter than the pre-determined path. Approximately 43% of the time, the experimental length was shorter than the expected length, and 57% of the time, the experimental length was longer. As a result, the accumulative position error, W, may grow or shrink depending on whether the error is additive or subtractive. 

**Figure 6:**
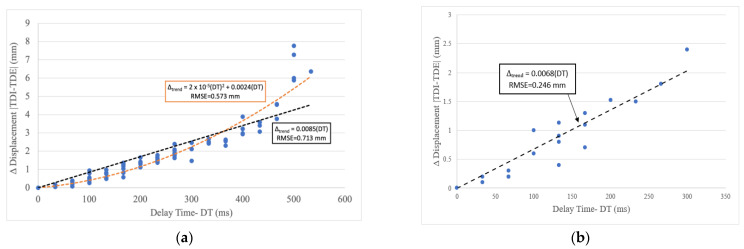
Position error with delay time using the (**a**) WLAN configuration, and (**b**) VLAN configuration.

Since tele-robotic systems need to be accurate and reliable, it is important to obtain a relationship between delay-time and end-effector position error. The accumulated data from all five trajectories (see Figure 5) were used to obtain trend lines, as seen in Figure 6a. 

The collected data revealed a maximum displacement error of 7.77 mm with a corresponding time delay of 500 milliseconds. The minimum displacement error was found to be 0.05 mm with a time delay of 33 ms. The median and mean displacement errors were found to be 1.40 mm and 1.85 mm, respectively. Statistical analysis was employed on the experimentally obtained samples to determine a model that relates displacement variation and time delay.

Root Mean Square Error (RMSE) was used to select the correct equation order that describes the model. The equation for RMSE is as follows:

(4) $$\mathrm{RMSE}=\sqrt{\frac{\sum {\left(\Delta -\Delta_{trend}\right)}^{2}}{N_{samples}}}$$

where ∆ is the displacement from experiments, $\Delta_{trend}$ is the displacement found with the trend equation, and $N_{samples}$ is the number of samples.

A maximum RMSE of 0.6 mm was established as the reference limit, which is 10% of the displacement error corresponding to the maximum *DT*. As shown in Figure 6a, the second-order trendline presents a smaller RMSE compared to the linear trendline.

From this analysis, it was identified that there is a quadratic relation between the delay and the displacement variation. This relation is described by the following equation:

(5) $$\Delta_{trend}=2*10^{-5}{\left(DT\right)}^{2}+0.0024\left(DT\right)$$

Therefore, an increase in delay time may significantly increase position error. Note that if the delay time and displacement error have a quadratic relationship, this would account for a reduction in Pearson’s correlation coefficient since the coefficient assumes their relationship is linear. 

### 3.3. VLAN Experiment Results

Experiments to measure the effect of changing to a VLAN configuration were performed using the same procedure as in the previous experiment. However, for this experiment, each sequence was repeated once, with a total of 18 movements. As before, both the time delay and corresponding position error were measured. The experimental results are plotted in Figure 6b; in contrast to Figure 6a, the newly reported data had a smaller range of both time delay and end-effector’s position error. The data obtained using the improved network resulted in a maximum delay of around 300 ms and a maximum position error of 2.4 mm.

The same statistical analysis was applied to the new experimental data. For this experiment, the same referential RMSE of 0.6 mm was selected to determine the model accuracy. As shown in Figure 6b, the improved network was sufficiently modeled by a linear trendline. It is unknown whether this is because a smaller range of delay times was measured or because the change in the network altered the delay–error relationship. This relation is described by the following equation:

(6) $$\Delta_{trend}=0.0068\left(DT\right)$$

As before, the overall communication behavior between the two networks was measured using the software. Compared to the WLAN configuration, the VLAN system showed a significant improvement with most measured delay values found to be between 100 ms and 200 ms. 

### 3.4. Discussion

Two different master-slave communication channels were tested to identify how delay alters the displacement accuracy of the MSK robot. First, the MSK system was studied using a WLAN setup. An analysis of collected data demonstrated that using a wireless network presents critically high delay values that alter the system’s operation. When the delay time increased, the position error increased significantly. As hypothesized, the VLAN performed better than the original WLAN configuration [20]. Overall, the VLAN experiments showed a lower latency and a better position accuracy.

Comparing both network options regarding maximum delay values, the VLAN achieved a reduction of about 200 ms compared to the WLAN. A similar situation occurred in terms of the end-effector’s position displacement variation, where the proposed improved network reduced the maximum position error by 70% to 2.4 mm. This accuracy has been found to be sufficient for ultrasound imaging [5]. Therefore, this experiment verifies that the network configuration significantly affects delay, and a VLAN would typically be more appropriate for teleoperation than a WLAN.

From the statistical analysis of the collected data, the improved communication network presented a linear trendline while the usual network revealed a quadratic trendline; this means a WLAN presents larger and faster displacement errors compared to the proposed improvement. This study has demonstrated that delay does not only reduce the responsiveness of the system, but also corresponds to worse physical behaviors such as position error. Furthermore, the growth in position error may increase with network delay as seen with the WLAN experiment. While delay is already considered undesirable, this further justifies steps to reduce or mitigate it. Designers should consider increasing the weight of delay mitigation as the measured delay increases. Users should be aware that the robot tested is mostly a cartesian robot with three prismatic joints and one rotary joint. If a robot with more rotary joints is being built, delay can affect performance more substantially because of the complications of rotary joints (more complicated inverse kinematics).

This work is a step forward in investigating control strategies for teleoperated manipulators. The experiments performed considered the effects of latency on position accuracy using a WLAN and VLAN for communication. Future work will involve measuring the user experience as a metric while controlling several variables such as programmed delay, signal frequency, signal size, network protocol, and network speed. Additional network configurations such as a VPN or wired LAN should also be considered. Finally, an optimal control strategy for tele-ultrasound should be considered. 

## 4. Conclusions

Teleoperated robots are valuable assets, which can be applied to a wide range of industries. However, as they are being used for more sensitive applications, such as medical diagnostics, it is important to improve the reliability of these systems. One notable source of concern is the time delay that occurs within the communication channel between the operator and the device.

The goal of this study was to measure the time delay in a teleoperated system, such as the MSK robot, evaluate its effects on the motion of the robot, and investigate a remedy for performance improvement. The time delay was measured through slow-motion video analysis and network analysis software. The robot end-effector was moved in pre-defined paths, and the end-effector’s position was compared with an ideal path with no delay.

By analyzing the experimental results, it was found that with the WLAN setup, the teleoperated MSK robot experienced a significant time delay with a maximum of around 500 ms. As the time delay increased, the end-effector position’s error increased approximately with a quadratic trend. The maximum end-effector position’s error was calculated to be 7.8 mm. With a VLAN setup, the average time delay was reduced by about 200 ms compared with the WLAN setup. The maximum end-effector position error was calculated to be about 2.4 mm for the VLAN setup.

## Figures and Tables

**Table 1:** Average position error and delay time of trajectory 5.

	TDI (mm)	TDE (mm)	∆ (mm)	W (mm)	DT (ms)
AB	30	30.24	0.24	1.42	206.8
BC	70	69.88	−0.12	3.42	180.2
CD	50	49.24	−0.76	3.34	233.2
DE	85	87.92	2.92	4.82	313.4

**Figure A1:**
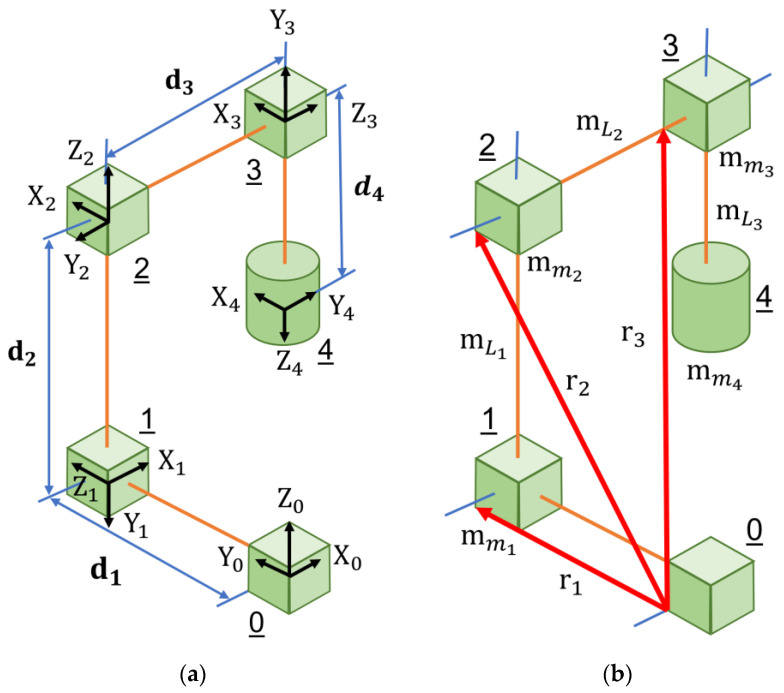
(**a**) Frames of the MSK manipulator, (**b**) Mass and position vector of the MSK manipulator.

**Figure A2:**
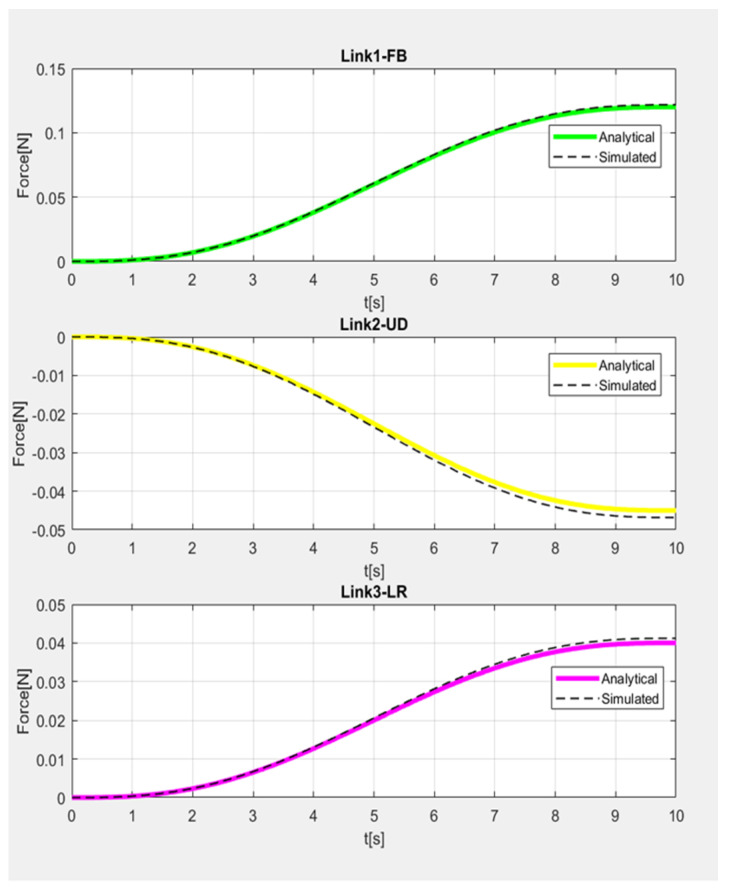
MSK robot; analytical vs. simulated joint forces; top- in the forward−backward (*y*-axis), middle- up−down (*z*-axis), and bottom- is left−right directions (*x*-axis) respectively.

## Data Availability

Data available on request due to restrictions. The data presented in this study are available on request from the corresponding author.

## Appendix A. DH Parameters

This section deals with generating the analytical model of the manipulator. The first step to create this model is to define the frames of reference and modified Denavit–Hartenberg (DH) parameters [21].

The schematic representation of the manipulator can be seen in Figure A1. A fixed frame {0} is set on the base. It is located at the end of the ball screw shaft, which drives the motion of the forward-back (FB) support. Frame {1} is also located on the same ball screw as frame {0} but directly below the ball screw shaft driving the left-right (LR) support. Frame {2} is located above frame {1} and along the shaft driving the LR motion. Frame {3} is located at the center of the ball screw nut which is driving the LR support. Frame {4} is located at the end of the probe. Prismatic joints are represented by cubes while the revolute joint is represented by a cylinder.

Based on Figure A1a, the DH parameters are shown in Table A1 Four variables are considered to derive the modified DH parameters [21]. These variables are as follows:

- *α_i_*, the measured angle between Z*_i_* and Z*_i_*_+1_ (about axis X_i_)
- *a_i_*, the measured distance between Z*_i_* and Z_i+1_ (along axis X_i_)
- *d_i_*, the measured distance between X*_i_*_−1_ and X*_i_* (along axis Z_i_)
- θ_i_, the measured angle between X*_i_*_−1_ and X_i_ (about axis Z_i_)

**Table A1:** Denavit–Hartenberg parameters.

Frame {*i*}	α*_i_*_−1_	*a_i_*_−1_	θ*_i_*	d*_i_*
1	−90°	0	0	d_1_
2	90°	0	90°	d_2_
3	90°	0	0	d_3_
4	−90°	−a_3_	θ_4_	−d_4_

## Appendix B. Kinematic Analysis

Forward kinematics obtain the end-effector position and orientation compared to the base of the manipulator. Using the DH parameters, general transformation matrices (Tii−1) can be derived:

(A1) Tii−1=Rii−10  0  0Pii−11

where Rii−1 is the rotational matrix from frame *i*−1 to *i*, and Pii−1 is the position of the base of frame *i* with respect to frame *i*−1.

The modified DH parameters always apply rotation in the same order: first about the *x*-axis, then about the *z*-axis. Therefore, we can find a general transformation matrix:

(A2) Tii−1=cosθi−sinθi0ai−1sinθicosαi−1cosθicosαi−1−sinαi−1−sinαi−1disinθisinαi−1cosθisinαi−1cosαi−1cosαi−1di0001

The transformations from frame {0} to frame {4} are:

(A3)
T10=1000001d10−1000001

(A4)
T21=0−10000−1−d210000001

(A5)
T32=100000−1−d301000001

(A6)
T43=cosθ4−sinθ40−a3001−d4−sinθ4 −cosθ4000001

Thus, the total transformation matrix is:

(A7)
T40=T10 T21 T32 T43

(A8)
T40=−sinθ4−cosθ40d3cosθ4−sinθ40d1−a300−1d2−d40001

The end-effector (probe) position in the *x*, *y*, and *z* directions relative to the base are described as follows:

(A9) $$px=d_{3}$$

(A10) $$py=d_{1}-a_{3}$$

(A11) $$pz=d_{2}-d_{4}$$

Since the three prismatic joints are orthogonal and the revolute joint is at the end-effector, the position can be confirmed through the geometric method as well. Note that parameters *d_i_* are variable for prismatic joints 1, 2, and 3 but fixed for revolute joint 4.

## Appendix C. Dynamic Analysis

It is now possible to conduct a dynamics analysis of the MSK to determine the joint forces. The derived analytical joint forces are then compared with a simulated model to verify accuracy.

The analytical model was determined using the Newton–Euler iterative algorithm, which calculates velocity and acceleration as it propagates from the base to the end-effector. It then determines the joint forces and torques from the end-effector to the base [21,22]. The dynamic model was also verified using the Lagrange approach. According to the Lagrange equation, the motion of each DOF, *i*, can be described as:

(A12) $$\frac{d}{dt}\left(\frac{\partial T}{\partial {\dot{x}}_{i}}\right)-\frac{\partial T}{\partial x_{i}}+\frac{\partial U}{\partial x_{i}}=Q_{i},\;i=1,2,3,4$$

where *T* is the system kinetic energy, *U* is the system potential energy, $Q_{i}$ are the external forces, while ${\dot{x}}_{i}$ and $x_{i}$ are the generalized velocity and position for each degree of freedom, respectively [12].

The velocity vector, ${\dot{r}}_{i}$, of joints 1, 2, and 3 can be found from position vectors, as follows:

(A13) $${\dot{r}}_{1}^{2}={\dot{d}}_{1}^{2}$$

(A14) $${\dot{r}}_{2}^{2}={\dot{d}}_{1}^{2}+{\dot{d}}_{2}^{2}$$

(A15) $${\dot{r}}_{3}^{2}={\dot{d}}_{1}^{2}+{\dot{d}}_{2}^{2}+{\dot{d}}_{3}^{2}$$

The kinetic energies for each DOF are as follows:

(A16) $$T_{1}=\left(m_{L_{1}}+m_{m_{2}}\right){\dot{d}}_{1}^{2}+\frac{1}{2}I_{m_{1}}k_{r_{1}}^{2}{\dot{d}}_{1}^{2}$$

(A17) $$T_{2}=\frac{1}{2}\left(m_{L_{2}}+m_{m_{3}}\right)\left({\dot{d}}_{1}^{2}+{\dot{d}}_{2}^{2}\right)+\frac{1}{2}I_{m_{2}}k_{r_{2}}^{2}{\dot{d}}_{2}^{2}$$

(A18) $$T_{3}=\frac{1}{2}\left(m_{L_{3}}+m_{m_{4}}\right)\left({\dot{d}}_{1}^{2}+{\dot{d}}_{2}^{2}+d_{3}^{2}\right)+\frac{1}{2}I_{m_{3}}k_{r_{3}}^{2}{\dot{d}}_{3}^{2}$$

(A19) $$T_{4}=\frac{1}{2}I_{m_{4}}{\dot{\theta }}_{4}^{2}$$

The notation $k_{r_{i}}$ is the gear ratio for the motor driving link *i*. Values $m_{L_{i}}$ and $m_{m_{i}}$ refer to the mass of link and joint *i*, respectively, while $I_{m_{i}}$ refers to the mass moment of inertia of the motors with respect to their axes of rotations. The total kinetic energy is:

(A20) $$\begin{array}{c} T=\frac{1}{2}\left(m_{L_{1}}+m_{m_{2}}+m_{L_{2}}+m_{m_{3}}+m_{L_{3}}+m_{m_{4}}\right){\dot{d}}_{1}^{2}+\frac{1}{2}\left(I_{m_{1}}k_{r_{1}}^{2}{\dot{d}}_{1}^{2}\right)\\ \;\;\;\;\;\;\;\;\;\;+\frac{1}{2}\left(m_{L_{2}}+m_{m_{3}}+m_{L_{3}}+m_{m_{4}}\right){\dot{d}}_{2}^{2}+\frac{1}{2}\left(I_{m_{2}}k_{r_{2}}^{2}{\dot{d}}_{2}^{2}\right)\\ \;\;\;\;\;\;\;\;\;\;+\frac{1}{2}\left(m_{L_{3}}+m_{m_{4}}\right){\dot{d}}_{3}^{2}+\frac{1}{2}\left(I_{m_{3}}k_{r_{3}}^{2}{\dot{d}}_{3}^{2}\right)+\frac{1}{2}\left(I_{m_{4}}{\dot{\theta }}_{4}^{2}\right) \end{array}$$

The potential energy of the system was assumed to only be derived from the effects of gravity and is:

(A21) $$U=\left(m_{L_{2}}+m_{m_{3}}+m_{L_{3}}+m_{m_{4}}\right)gd_{2}$$

where *g* is the gravitational acceleration. Finally, using the Lagrange equation, the torque applied to the revolute joint and the forces applied to the prismatic joints are derived as follows:

(A22) $$F_{1}=\left(m_{L_{1}}+m_{m_{2}}+m_{L_{2}}+m_{m_{3}}+m_{L_{3}}+m_{m_{4}}+I_{m_{1}}k_{r_{1}}^{2}\right){\ddot{d}}_{1}$$

(A23) $$F_{2}=\left(m_{L_{2}}+m_{m_{3}}+m_{L_{3}}+m_{m_{4}}+I_{m_{2}}k_{r_{2}}^{2}\right){\ddot{d}}_{2}+\left(m_{L_{2}}+m_{m_{3}}+m_{L_{3}}+m_{m_{4}}\right)$$

(A24) $$F_{3}=\left(m_{L_{3}}+m_{m_{4}}+I_{m_{3}}k_{r_{3}}^{2}\right){\ddot{d}}_{3}$$

(A25) $$\tau_{4}=\left(I_{m_{4}}\right){\ddot{\theta }}_{4}$$

where $F_{i}$ and $\tau_{4}$ are the force and torque to drive joints *i* = 1, 2, 3, and 4, respectively. The dynamics equations obtained using the Lagrange approach were verified using the Newton–Euler iterative algorithm.

The trajectory used to compare the two models used a cubic displacement function that moved in each degree of freedom sequentially. The acceleration and deceleration portion of each segment lasted 10 s each. Both the end-effector position and joint forces were compared. Figure A2 shows the joint forces during acceleration for the analytical and simulated models. Note that Figure A2 shows the forces for the acceleration portion only. Figure A2 top shows the joint force applied by the link moving forwards and backward (FB) in the *y*-axis. Figure A2 middle shows the joint force applied by the link moving up and down (UD) in the *z*-axis. Figure A2 bottom shows the joint force applied by the link moving left and right (LR) on the *x*-axis. As shown, both the position and joint forces were relatively accurate.
